# Operational pollen classification using digital holography and fluorescence

**DOI:** 10.1007/s10453-025-09882-w

**Published:** 2025-09-20

**Authors:** Benoît Crouzy, Marie-Pierre Meurville, Bernard Clot, Sophie Erb, Maria Lbadaoui-Darvas, Fiona Tummon, Gian Lieberherr

**Affiliations:** 1 Surface Measurements, MeteoSwiss, Chemin de l’Aérologie, 1530 Payerne, Switzerland; 2 Environmental Remote Sensing Laboratory (LTE), École Polytechnique Fédérale de Lausanne, Station 1, 1015 Lausanne, Switzerland; 3 Laboratory of Atmospheric Processes and their Impacts (LAPI), École Polytechnique Fédérale de Lausanne, Station 1, 1015 Lausanne, Switzerland; 4 Institute of Chemical Engineering Sciences Hellas, Foundation for Research and Technology (FORTH/ICE-HT), 26504 Hellas, Patras, Greece

**Keywords:** Pollen monitoring, Machine learning, Real-time, Digital holography, Fluorescence, Automatic identification, Airflow cytometry

## Abstract

This note introduces the newly developed MeteoSwiss operational pollen classification model based on digital holography and induced fluorescence measurements. A targeted selection of curated training datasets together with a revised model architecture result in considerable improvements compared to previous operational model. The new classification model, which has been trained specifically for Switzerland, is provided openly for use in a standard format for machine learning interoperability. In addition to the description of the new classification model, we motivate the need for this development by presenting the most significant issue met during the first 5 years of operation of the Swiss automatic pollen monitoring network.

Pollen monitoring networks around the world have begun a radical transition from manual monitoring (Hirst, 1952) to the use of automatic systems (see, for example, Crouzy et al. (2016), Sauvageat et al. (2020), Šauliene et al. (2019), Oteros et al. (2015) and Buters et al. (2024) for a review). Over the past 7 years across Europe, the number of automatic pollen monitoring systems has increased from less than ten devices used for research to over 60 devices, many of which are used in operational monitoring networks. One example of such automatic systems is the SwisensPoleno (Swisens AG), an airflow cytometer that measures aerosols using digital holography and fluorescence. Details on the holographic technique used can be found in Sauvageat et al. (2020) and references therein. A proof-of-concept phase, testing three SwisensPolenos in parallel, was carried out in Switzerland for the full 2020 pollen season. A year later, a large instrument intercomparison campaign was held in Germany (Maya-Manzano et al., 2023) comparing nine different types of automatic pollen monitors. The results from the proof-of-concept and the intercomparison showed that the SwisensPoleno performed well. Consequently, over the 2021–2022 period, the Federal Office of Meteorology and Climatology MeteoSwiss (‘MeteoSwiss’ hereafter) installed 15 such instruments across Switzerland following a public tender. Since January 2023 this network, the SwissPollen network, has been operational using a pollen classification algorithm based on holographic images only (Sauvageat et al., 2020). Real-time observations are publicly available, and are also directly integrated into the MeteoSwiss numerical forecasting system, resulting in significantly improved forecasts (Adamov and Pauling, 2023). In order to foster collaboration and increase the reproducibility of aerobiological studies, it is essential to document the monitoring methods and machine learning models. This note shall provide such reference for the Swiss pollen monitoring network.

**Fig. 1:**
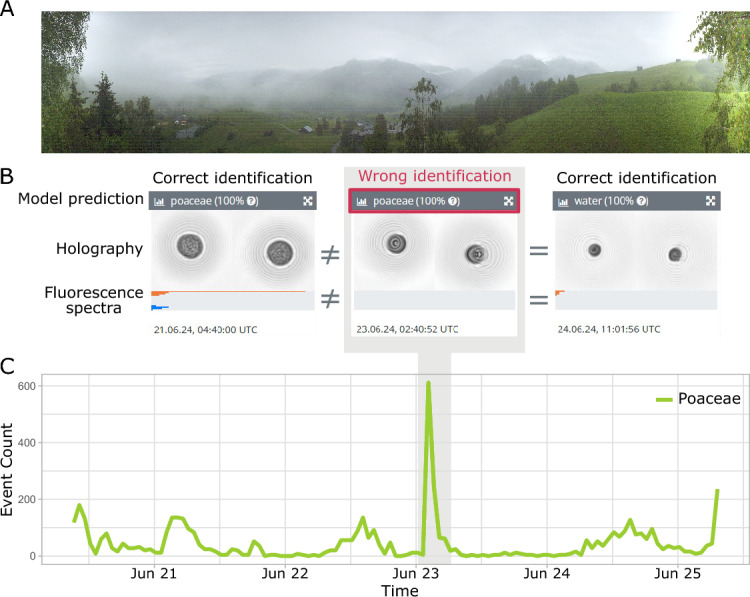
Example of a timeseries from the SwissPollen monitoring site in Davos, Switzerland, highlighting the problem of misclassification of water droplets. **A** Photo of the Davos site from 23 June 2024 at 06:30 UTC. **B** From left to right: example of individual event identified by the algorithm as grass pollen (Poaceae) [true positive], water droplet misidentified as grass pollen [false positive], and correctly identified water droplet [true negative]. Each event is described by two holograms (200 $\times$ 200 pixels, greyscale) and measurements from 13 fluorescence channels. **C** Timeseries of aggregated hourly counts of aerosols classified as grass pollen between the 20–25 June 2024. The peak highlighted in grey is mostly composed of water droplets misclassified as grass pollen

During the first years of operational experience, several issues have been identified. These include false detection of various taxa outside their respective main pollen seasons as well as device-to-device comparability. Specifically, as shown in Fig. 1, water droplets between 10 and 100 μm in size are present under saturated atmospheric conditions (e.g. fog or heavy precipitation events) and can be confused with the smooth round grass pollen grains or sometimes other pollen types. This is particularly problematic, as grass pollen is the main allergenic taxon in Switzerland (Wüthrich et al., 2009). The supervisor approach introduced by Crouzy et al. (2022) showed some level of success in handling out-of-season false-positive classifications. However, in-season false-positive detection remains an issue that has so far been addressed by operational quality control procedures (manual and automatic correction). Another issue is related to species within the Betulaceae family that are difficult to distinguish from each other when using only digital holography (Sauvageat et al., 2020). To address these problems, new classification algorithms that also take fluorescence measurements as input have been developed. Limited controlled experiments have proven the potential for fluorescence to resolve confusions between certain allergenic pollen taxa (Erb et al., 2024).

**Fig. 2:**
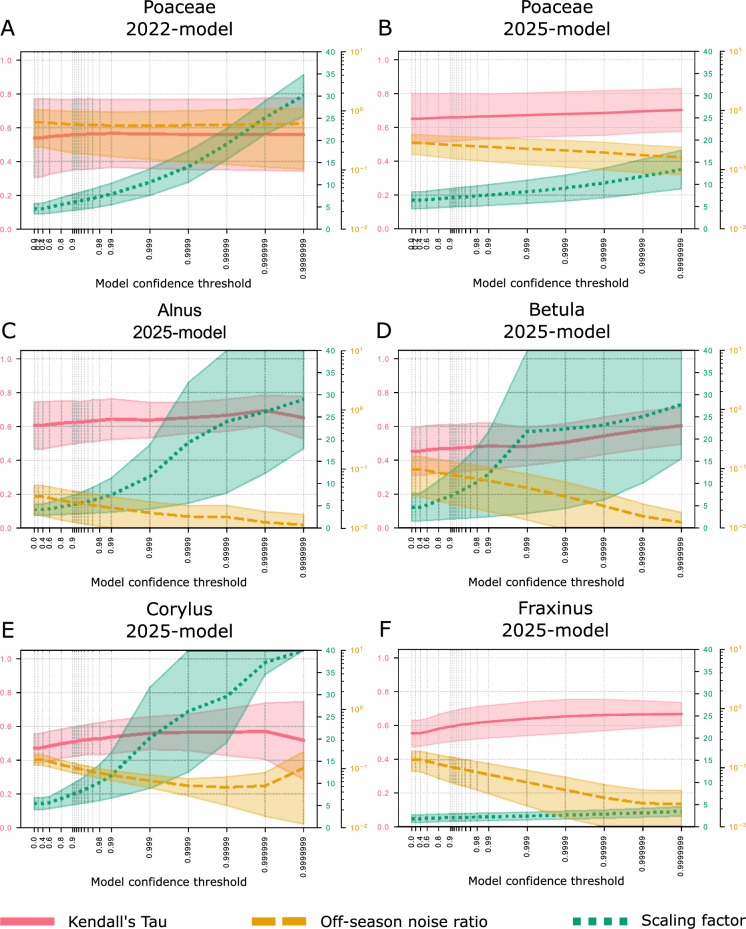
Dependence of the performance metrics on the model confidence threshold for five classes with the proposed 2025 model and one class with the former 2022 model. The three metrics are computed for **A** grass (Poaceae) using the 2022 model and the 2025 model, for **B** grass (Poaceae), **C** *Alnus* sp., **D** *Betula* sp., **E** *Corylus* sp. and **F** *Fraxinus* sp., over different five sites. The red, green, and orange lines illustrate the averaged Kendall’s Tau, the scaling factor, and the off-season noise ratio, respectively. The shading delimits the minimum and maximum values for each metric across the five sites

Following the work of Erb et al. (2024), we developed a new operational classification algorithm based on the following four principles: (1) It should flexibly handle situations where no fluorescence observations are available, since not all SwisensPoleno devices were (in the first years) equipped with fluorescence modules and not all events display a fluorescence signal above the noise threshold. This requirement can readily be accommodated by a two-branch neural network architecture able to provide classifications with and without fluorescence input. In order to avoid over-training linked to the fluorescence part of the signal we used for training a significant number of datasets without fluorescence and, on the datasets including fluorescence, we discarded 20% of the fluorescence signals during training. The fluorescence branch of the network includes few neurons in order to avoid over-complex fluorescence features (see GitHub model repository for details (MeteoSwiss biometeorology team, 2025)). (2) The algorithm must identify the main allergenic pollen taxa in Switzerland (Poaceae (grasses), *Betula* sp., *Corylus* sp., *Alnus* sp., *Fraxinus* sp., *Quercus* sp., and *Fagus sylvatica*). In addition to these taxa, several additional training datasets were included covering other pollen taxa present in significant quantities, as well as water droplets (a full list describing the training datasets can be found on GitHub (MeteoSwiss biometeorology team, 2025)). Non-biological particles are filtered out using the procedure described by Sauvageat et al. (2020) which relies on a deterministic morphological filter applied prior to the neural network. Water droplets training datasets were created from operational data during fog or rain events by manually filtering out unwanted particles based on morphology. (3) The architecture should be optimised for handling greyscale images as produced by the SwisensPoleno digital holography module. This led to the application of a simple, customised convolutional neural network rather than a pre-trained neural network optimised for RGB images as applied, for example, by Erb et al. (2024). The detailed network architecture is inspired by the VGG architecture similar to the one used in Sauvageat et al. (2020) and is presented in detail on GitHub (MeteoSwiss biometeorology team, 2025). Note that the model was trained from scratch and no pre-trained neural network was used. Finally, (4) the training datasets need to reflect the full diversity of characteristics of each pollen taxon. This can be assured by creating training datasets from different plants, locations and under different meteorological conditions (Erb et al., 2025). These datasets need to be manually cleaned to remove various artefacts such as aggregates and debris.

Here we briefly present results obtained using the new MeteoSwiss operational algorithm developed in 2025. The algorithm evaluation is performed for five sites during the 2024 pollen season (Luzern: 15 January–10 June, Neuchâtel: 11 January–1 June, Payerne: 3 May–1 September, Buchs: 16 January–14 September, and Basel: 12 January–4 May). Three metrics are calculated using parallel manual measurements from Hirst-type traps. The **Kendall’s Tau correlation** coefficient, which is less susceptible to outliers than the Pearson coefficient, quantifies the correlation between manual and automatic measurements. The **scaling factor** represents the ratio between manual and automatic measurements; the lower the scaling factor, the better the sampling using the automatic system. Finally, the **off-season noise ratio** serves as an indicator of the noise induced by false-positive detections of the classifier. It is defined as the ratio of the averaged daily pollen concentrations outside the defined pollen season to the averaged daily pollen concentrations during the season. The out-of-season period is defined when, over any sliding window of seven consecutive days, at least four days have average daily pollen concentrations below 20 particles/m³, based on the manual measurements. A low off-season noise ratio means fewer incorrect warnings to the public outside of the pollen season and indicates a better signal-to-noise ratio in general. All three metrics are presented as a function of the confidence threshold applied to the classifier output (Crouzy et al., 2022), which provides useful information to select the optimum confidence threshold. We do not use the supervisor approach (Crouzy et al., 2022) so as to provide an impartial assessment of the algorithms out of the main pollen seasons.

The two upper panel of Fig. 2 show how the new 2025 algorithm improves grass pollen classification. The average values for all three metrics are better than for the 2022 model. Depending on the selected threshold, the spread remains low, indicating acceptable coherence between the five sites. The increase of spread for higher confidence threshold in panel (B) of Fig. 2 highlights which confidence threshold should be selected as a trade-off optimising correlation while keeping sufficient sampling. Note that in an operational setup not too high confidence thresholds need to be used in order to ensure reproducibility of results. The lower panels of Fig. 2 show the same three metrics for the 2025 model for the four most relevant (Wüthrich et al., 2009) additional pollen taxa, *Alnus* sp., *Betula* sp., *Corylus* sp., and *Fraxinus* sp. in order to present model performance for aero-allergen monitoring and to assess device-to-device variability.

The high Kendall’s Tau correlations and low scaling factors indicate that the 2025 algorithm performs well, particularly given the limitations of manual measurements (Oteros et al., 2017). These results also show that the algorithm is capable of discriminating between taxa that are typically misclassified (Šauliene et al., 2019; Erb et al., 2024). Interestingly, for lower confidence thresholds all metrics are good, while limiting the spread across the five sites.

A more detailed evaluation and discussion of various classification algorithms is beyond the scope of this short note and will be presented in a companion paper. We provide however here a self-contained reference to allow new developments based on this model and to ensure reproducibility of studies based on data from the MeteoSwiss operational pollen monitoring network. The neural network architecture, as well as the confidence thresholds, corresponding scaling factors optimised for the Swiss network and training sets, are available on GitHub (MeteoSwiss biometeorology team, 2025). To facilitate reuse, the open ONNX format is used (Bai et al., 2019). This classification algorithm may serve as a benchmark for Switzerland to evaluate future algorithms. Following the introduction of the new model in the Swiss operational network, a reanalysis of previous raw data will provide consistent timeseries for climatological applications. However, it is important to note that the model should not be used operationally in environments without prior validation, as other pollen taxa or aerosol particles as well as other environmental conditions could affect the performance in unexpected ways.

## Data Availability

The neural network architecture, as well as the confidence thresholds, corresponding scaling factors optimised for the Swiss network and training sets, is available on GitHub (https://github.com/MeteoSwiss/swisspollen-models). To facilitate reuse, the open ONNX format is used.
